# Root microbiome dynamics favor slow-growth strategies during *Pinus* seedling development

**DOI:** 10.1093/ismeco/ycag018

**Published:** 2026-01-29

**Authors:** Chenyu Sun, Cong Wang, Huanhuan Zhu, Xiaoye Chen, Peilin Chen, Qiushi Li, Zheng Gao, Bo Yang, Lei Chen, Ningning Wang, Liangdong Guo, Cheng Gao

**Affiliations:** College of Life Sciences, Shandong Agricultural University, Taian, 271018, Shandong, China; State Key Laboratory of Microbial Diversity and Innovative Utilization, Institute of Microbiology, Chinese Academy of Sciences, Chaoyang District, 100101, Beijing, China; State Key Laboratory of Microbial Diversity and Innovative Utilization, Institute of Microbiology, Chinese Academy of Sciences, Chaoyang District, 100101, Beijing, China; College of Life Sciences, University of Chinese Academy of Sciences, Huairou District, 100049, Beijing, China; State Key Laboratory of Microbial Diversity and Innovative Utilization, Institute of Microbiology, Chinese Academy of Sciences, Chaoyang District, 100101, Beijing, China; College of Life Sciences, University of Chinese Academy of Sciences, Huairou District, 100049, Beijing, China; State Key Laboratory of Microbial Diversity and Innovative Utilization, Institute of Microbiology, Chinese Academy of Sciences, Chaoyang District, 100101, Beijing, China; College of Life Sciences, University of Chinese Academy of Sciences, Huairou District, 100049, Beijing, China; State Key Laboratory of Microbial Diversity and Innovative Utilization, Institute of Microbiology, Chinese Academy of Sciences, Chaoyang District, 100101, Beijing, China; College of Life Sciences, University of Chinese Academy of Sciences, Huairou District, 100049, Beijing, China; State Key Laboratory of Microbial Diversity and Innovative Utilization, Institute of Microbiology, Chinese Academy of Sciences, Chaoyang District, 100101, Beijing, China; College of Life Sciences, University of Chinese Academy of Sciences, Huairou District, 100049, Beijing, China; College of Life Sciences, Shandong Agricultural University, Taian, 271018, Shandong, China; School of Biological and Environmental Engineering, Jingdezhen University, Jingdezhen, 333400, Jiangxi, China; State Key Laboratory of Vegetation and Environmental Change, Institute of Botany, Chinese Academy of Sciences, Haidian District, 100093, Beijing, China; State Key Laboratory of Vegetation and Environmental Change, Institute of Botany, Chinese Academy of Sciences, Haidian District, 100093, Beijing, China; State Key Laboratory of Microbial Diversity and Innovative Utilization, Institute of Microbiology, Chinese Academy of Sciences, Chaoyang District, 100101, Beijing, China; College of Life Sciences, University of Chinese Academy of Sciences, Huairou District, 100049, Beijing, China; State Key Laboratory of Microbial Diversity and Innovative Utilization, Institute of Microbiology, Chinese Academy of Sciences, Chaoyang District, 100101, Beijing, China; College of Life Sciences, University of Chinese Academy of Sciences, Huairou District, 100049, Beijing, China

**Keywords:** seedling, genomic traits, temporal dynamic, nitrogen addition

## Abstract

Microbial functional trait dynamics during seedling development—a critical yet underexplored driver of forest ecosystem establish, develop, and stability—remain poorly understood. We investigated bacterial genomic traits dynamics in subtropical *Pinus massoniana* seedlings over a growing season. Leaf-associated bacteria showed minimal temporal shifts, whereas root-associated bacteria exhibited pronounced trends: average genome size increased (independent of nitrogen addition), whereas ribosomal RNA operon copy number (RRN) declined under ambient nitrogen, indicating a transition from fast-growing to slow-growing strategies. These trajectories reflect the differential turnover of later, nitrogen-insensitive taxa (e.g. large-genome, low-RRN *Bradyrhizobium*) relative to earlier, nitrogen-sensitive taxa (e.g. small-genome, high-RRN *Herbaspirillum*) during colonization and establishment from an aerial source onto a developing host. Additionally, we detected a discrepancy between the temporal dynamics of predicted nitrogen fixation potential and quantitative real-time PCR-based *nifH* quantification, underscoring the need for caution when interpreting prediction-based functional potentials. These findings identify trait-mediated assembly as a key driver of early root microbiome dynamics in pine seedlings and highlight the need for temporally resolved, ground-truthed functional inference when predicting ecosystem processes.

## Introduction

Tree seedlings are a cornerstone of forest ecosystems, playing pivotal roles in maintaining community diversity, stability, and long-term forest succession dynamics [1]. However, seedlings generally experience high mortality in forests due to resource limitations, predation, pathogen attack, and other factors [2–4]. Emerging evidence indicates that seedlings form a tightly integrated holobiont with their associated microbiomes [5]. This symbiotic relationship is bidirectionally shaped: seedlings actively structure their microbiome through compartment-specific differentiation (e.g. roots versus leaves) and developmental stage-dependent turnover [6], while the microbiome reciprocally influences seedling fitness through context-dependent benefits (e.g. nutrient mobilization) or costs (e.g. pathogenicity) [7]. Bacteria are often among the earliest microbial colonizers and are important during the early seedling development, contributing to plant growth, stress tolerance, disease resistance, and rhizosphere nutrient dynamics, including nitrogen cycling [8–10]. Deciphering the temporal dynamics of the seedling-associated bacterial microbiome is therefore essential for understanding the mechanisms underlying seedling survival and mortality [11].

Genomic traits are a valuable way to capture microbiome characteristics because they constitute the genetic basis of microbial activity and provide insight into how microbes respond and adapt to changes in resources and stress [12–14]. Genome size and ribosomal RNA operon copy number (RRN) are major genomic traits that influence bacterial function and environmental adaptation [15, 16]. Genome size is usually positively correlated with gene counts [17] and can therefore indicate functional versatility, which is important for microbial adaptation to stress and competition for resources [18, 19]. RRN is a genomic trait associated with bacterial maximum growth rate [18]. A higher RRN is linked to faster maximum growth and is prevalent in copiotrophic environments [20, 21], whereas a lower RRN may represent an conservatism strategy under resource-limited conditions [20, 22]. Several studies have shown that these genomic traits are influenced by environmental factors such as pH [16, 17, 23, 24], nutrient availability [16, 25], temperature [15, 26], oxygen levels, and salinity [27, 28]. However, the patterns and mechanisms underlying changes in microbial genome size and RRN during plant development remain poorly understood.

During seedling development, host selection may progressively shape associated microbial communities, leading to shifts in microbial genomic traits such as genome size and RRN [6, 29, 30]. Developing seedlings may favor microbes with larger genomes and lower RRN, traits commonly associated with greater functional versatility and slower growth, which can be advantageous in increasingly competitive and heterogeneous plant-associated environments [31]. Across four published datasets, later developmental stages generally show a decline in average RRN in most late-successional samples [32]. By contrast, average bacterial genome size exhibits divergent patterns: it increased slightly in the *Populus* phyllosphere, decreased slightly in *Arabidopsis*, was largely unchanged in apple flowers, and increased markedly in kelp [32]. Such variability may reflect differences in host traits, environmental context, study duration, or methodology. Most prior studies have focused on aboveground plant tissues, and much less is known about shifts in genomic traits among root-associated microbes. Fierer *et al*. [33] described community changes during plant development as “microbial succession,” and distinct nutrient conditions and abiotic stresses between roots and phyllospheres [34, 35] can shape distinct successional trajectories of their associated microbiomes. For example, recent studies show that the temporal dynamics of root-associated microbial community composition are more distinct from those of phyllosphere-associated microbes during plant development [29, 30], suggesting that changes in genomic traits of root-associated microbes may be more pronounced than those of phyllosphere-associated microbes. Notably, most of these studies emphasize broadleaf plants, even though conifers (e.g. *Pinus* spp.) dominate many subtropical and temperate forests [36]. For example, *Pinus massoniana* is an evergreen conifer widely distributed in Southeast China, yet the genomic traits of microbial communities associated with developing *P. massoniana* seedlings remain unknown. Here, we hypothesize that average bacterial genome size in the leaves and roots of *P. massoniana* increases during seedling development, whereas average RRN decreases, reflecting adaptation to a progressively more competitive and heterogeneous plant-associated environment.

Nitrogen (N) deposition has been one of the most widespread global environmental changes in recent decades, driven by intensified anthropogenic activities such as fossil fuel combustion, N fertilization, and the cultivation of N-fixing crops [37, 38]. N deposition is projected to continue increasing in the foreseeable future, with the tropics and subtropics becoming hotspots [39]. Recent studies suggest that N deposition may contribute to range shifts in forest plants [40], and one possible mechanism is that N deposition affects plant–microbe interactions [41, 42]. On the one hand, N deposition can directly influence microbes via nutrient enrichment and soil acidification [43, 44]. On the other hand, it can disrupt nutrient balance—especially by inducing phosphorus limitation—thereby altering plant nutrient demand and indirectly shaping microbial communities [45]. These environmental changes may influence microbial genomic traits, as microbial life-history strategies shift in response to N-induced alterations in resources and stress [46]. Accordingly, we hypothesize that N deposition will alter the temporal dynamics of genome size and RRN in plant-associated microbes as a result of N-induced changes in resource availability and stress [41, 45].

To test these hypotheses, we tracked the temporal dynamics of average genome size and RRN in roots and leaves during one growing season of initially sterilized *P. massoniana* seedlings, with and without N addition. As a reference, we also tracked the temporal dynamics of these two genomic traits in airborne bacteria, which served as the source of seedling-associated microbes in this study. We conducted a germination trial of *P. massoniana* seeds under controlled greenhouse conditions. Following successful seedling establishment, the young plants were transferred to a subtropical forest ecosystem. Concurrently, we deployed airborne microbial samplers to collect airborne bacteria in the surrounding environment. Both plant and aerobiological samples were collected monthly over a 7-month period from June to December. We used 16S ribosomal RNA (rRNA) gene amplicon sequencing to assess bacterial taxonomic diversity and composition, and we estimated average genome size and RRN based on the sequencing data using the Genome Taxonomy Database (GTDB, v202) and the Ribosomal RNA Operon Copy Number Database (rrnDB, v5.8).

## Materials and methods

### Study site

This study was conducted in the Gutianshan National Nature Reserve (29°08′18″-29°17′29″N, 118°02′14″-118°11′12″E), Suzhuang Town, Kaihua County, Zhejiang Province, China. The reserve covers ~81 km^2^ and lies in a subtropical monsoon climate, with a mean annual temperature of 15.38°C and mean annual precipitation of 1964 mm [47–49]. *Castanopsis eyrei*, *Schima superba*, and *P. massoniana* are the most common canopy tree species in the area [49]. We focused on *P. massoniana* because it is a pioneer species in both primary and secondary succession in this region [50].

### Experimental design

We used initially sterile *P. massoniana* seedlings. Seedlings were germinated from seeds in the greenhouse at Jingdezhen University, located ~200 km from the Gutianshan National Nature Reserve. The growth substrate consisted of forest topsoil and river sand (1:1, v/v) and was autoclaved twice (121°C, 2 h each), 24 h apart. Commercial *P. massoniana* seeds were purchased from Greenland Seed Industry. Well-filled, fresh seeds were surface-sterilized with 0.5% KMnO₄ solution for 2 h, thoroughly rinsed with sterile water, soaked at 40°C for 24 h, and incubated at 25°C with sterile-water rinses twice daily. Once the seeds had cracked and the white embryo was visible, they were sown in the sterilized substrate on 22 March 2022. Throughout the seedling phase, sterile water was used to standardize germination and minimize microbial contamination, and pots were covered. On 6 June 2022, when seedlings had ~30 needle leaves, they were transported by truck to the field site at Gutianshan National Nature Reserve.

The field site consisted of three open sites (~50 m apart) lacking canopy cover. At each site, a 3 m × 2.5 m plot was established using 0.5 m-high wire mesh fencing to house airborne microbial collectors and *P. massoniana* seedlings. In each plot, we placed 288 potted seedlings, of which 117 pots received N addition. Nitrogen addition was applied at 2.5 kg N ha^−1^ month^−1^ (30 kg N ha^−1^ yr^−1^) immediately after each sampling to simulate long-term elevated deposition. Given ambient deposition of ~30 kg N ha^−1^ yr^−1^ at Gutianshan [51], this treatment approximately doubled total N input.

**Figure 1:**
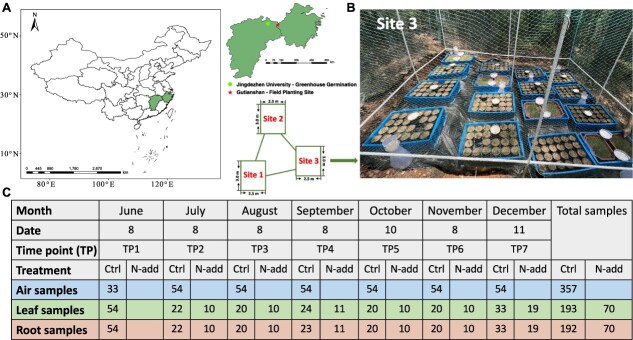
Experimental design; (A) location of the greenhouse (circle) and field planting site (star), and a schematic showing the distribution of the three field planting site; (B) arrangement of *Pinus massoniana* seedlings and airborne microbial samplers at the field experimental site; (C) number of samples collected at different time points (TP1–TP7), compartments (air, leaf, and root), and treatments (control, and N addition) at the field experimental site; Ctrl, control (without N addition); N-add, N addition; the basemap data were obtained from ChinaAdminDivisonSHP v23.01.04 (https://zenodo.org/records/7503181) and visualized in ArcMap 10.8.

We also established 18 passive, filtration-based airborne microbial samplers (Fig. 1B). Each sampler consisted of a funnel (9 cm diameter) and a circular filter paper (15 cm diameter; double-ring quantitative slow filter paper, mean pore size 2 μm) secured with a magnetic clamp. The funnel was positioned centrally within the *P. massoniana* pots, at the same height as the potted plants.

### Field sampling

In 2022, root, leaf, and air-filter samples were collected monthly over a 7-month period (8 June, 8 July, 8 August, 8 September, 10 October, 8 November, 11 December). At each sampling, 10 pots without N addition and five pots with nitrogen addition were randomly harvested from each site. Seedlings were manually removed from pots and gently shaken to remove soil aggregates [52]. Seedlings were separated at the shoot-root junction using sterile scissors; shoots and roots were placed in sterile bags. At site 1, seedlings were collected only on 8 June and 11 December due to the limited number of surviving seedlings. Filter paper was removed into sterile ziplock bag; the holder was cleaned with 75% ethanol followed by sterile water, and new filter paper was installed for the next sampling. In total, 262 root samples, 263 leaf samples, and 357 air samples were collected (Fig. 1C). Root, leaf, and air samples were immediately placed on ice and transported to the laboratory, then stored at −80°C until DNA extraction.

### DNA extraction

Microbial community DNA from leaves and roots was extracted using the modified cetyltrimethylammonium bromide (CTAB) method [53]. Frozen roots were transferred to a 20 ml tube containing 15 ml sterile, chilled phosphate buffer saline (PBS), vortexed to remove attached soil, and homogenized at 6.0 m s^−1^ for 40 s, three times, in a FastPrep-24 instrument (MP Biomedicals, Illkirch, France), following the manufacturer’s instructions [54], with brief cooling between cycles. Samples were then incubated at 65°C for 60 min with occasional gentle swirling. Subsequent steps followed the modified CTAB protocol [53].

Airborne bacterial DNA was extracted with the DNeasy PowerSoil Pro Kit (Qiagen, Germany). Each filter paper sample was cut into ~2 cm × 2 cm pieces and transferred to a sterile 100 ml Erlenmeyer flask containing 40 ml preheated 4 × Tris-EDTA (TE) buffer (40 mM Tris–HCl, 4 mM ethylenediaminetetraacetic acid (EDTA), pH 8.0) at 70°C and 1 μl IGEPAL CA-630. Flasks were shaken on an orbital shaker for 2 h. The solution was transferred to a 50 ml tube and centrifuged at 11000 × g for 15 min; the supernatant was discarded. The pellet was resuspended to a final volume of 4 ml by vortexing, divided into two 2 ml tubes, and centrifuged at 14000 × g for 5 min. Supernatants were discarded. Each pellet was resuspended to 750 μl, combined into one microcentrifuge tube, and centrifuged at 14000 × g for 5 min. The supernatant was discarded, leaving ~400 μl of resuspended pellet. Enriched microbial suspensions were stored at −80°C until DNA extraction per the kit protocol, modified with a 40 s homogenization at 6.0 m s^−1^ (MP FastPrep-24 5G) and a 10-min water bath at 70°C.

### Amplicon sequencing and bioinformatics

DNA concentrations and quality were assessed with a NanoDrop 2000 spectrophotometer (Thermo Fisher Scientific, Waltham, MA, USA). DNA was then used for polymerase chain reaction (PCR) amplification and sequencing to profile bacterial communities. The V4 region of the 16S rRNA gene was amplified with primers 515F (5′-GTGYCAGCMGCCGCGGTAA-3′) and 806R (5′-GGACTACNVGGGTWTCTAAT-3′) [55]. PCR cycling conditions were: 98°C for 30 s; 35 cycles of 98°C for 10 s, 56°C for 5 s, and 72°C for 40 s; and a final extension at 72°C for 1 min. PCR products were purified using a gel purification kit (Axygen), and concentrations were determined with a Qubit 2.0 fluorometer (Life Technologies, CA, USA). Illumina adapters and indices were added according to the manufacturer’s instructions, and libraries were sequenced on an Illumina NovaSeq 6000 (Shanghai Personal Biotechnology Co., Ltd).

Raw sequences were quality filtered and demultiplexed in QIIME 2 (v2023.7) [56]. High-quality reads were processed in USEARCH v11.0.667 for dereplication and chimera removal [57]. Non-chimeric sequences were clustered into operational taxonomic units (OTUs) at 97% similarity using cluster_otus [58, 59]. Representative OTU sequences were taxonomically assigned with the SILVA v138.1 database [60]. Reads for each sample were rarefied to a uniform sequencing depth of 2000 reads per sample, and the resulting rarefied OTU tables were used for downstream analyses.

### Quantification of the *nifH* gene

The *nifH* gene copy number in each root sample was quantified by quantitative real-time PCR (qPCR) using primers PolyF (5′-TGCGAYCCSAARGCBGACTC-3′) and PolyR (5′-ATSGCCATCATYTCRCCGGA-3′) [61, 62]. qPCR reactions (20 μl) contained 10 μl of 2× SYBR Green Master Mix (Vazyme, China), 0.4 μl of each primer (10 μM), 8 μl of template DNA, and 1.2 μl of nuclease-free water. Amplification was performed on a LightCycler 480 II (Roche, Switzerland) with 95°C for 5 min, followed by 40 cycles of 95°C for 15 s, and 60°C for 30 s. Fluorescence was collected at the end of each cycle. A melt-curve analysis was performed to confirm amplification specificity. Standards were generated by serial dilution of plasmids containing a known *nifH* insert [63]. Initial *nifH* copy numbers were calculated from the standard curve based on the threshold cycle (Ct) values. Each sample was measured in triplicate, and *nifH* abundance was expressed as copies ng^−1^ DNA.

### Genomic traits and gene functions

Bacterial genome size and RRN were predicted by matching representative 16S rRNA gene sequences of each OTU to the GTDB v202 [64] and the rrnDB v5.8 [22, 65, 66], respectively. Previous studies show that average genome size predicted from amplicon data is highly consistent with metagenomic estimates in ecological analyses, supporting amplicon-based genomic trait annotation [16, 17, 67, 68]. Metadata and FASTA files for representative genomes were downloaded from GTDB [64] and rrnDB [22, 65, 66]. Reference databases were constructed with makeblastdb (BLAST v2.16.0), and amplicon sequences were aligned with Basic Local Alignment Search Tool (BLAST) v2.16.0 to obtain genome accession IDs. Genome size and RRN values were retrieved by matching accession IDs to the corresponding metadata. To assess robustness, we reprocessed reads using the DADA2 pipeline [69] within QIIME 2 [56] to generate amplicon sequence variants (ASVs) and repeated database matching against GTDB and rrnDB.

Trait assignments were retained only for alignments with identity ≥95% and coverage ≥95%. Under these criteria, the relative abundance of OTUs included in genome-size and RRN analyses ranged from 73.32% to 94.14% and from 54.87% to 85.02%, respectively (Supplementary Fig. S1). Dominant genera exhibited low coefficients of variation for genome size and RRN estimates in their reference datasets (Supplementary Figs S2 and S3). For each sample, average genome size and RRN were calculated as abundance-weighted means based on OTU read counts.

Bacterial functional potential was predicted with the phylogenetic investigation of communities by reconstruction of unobserved states (PICRUSt2) [70] using the OTU abundance table and representative sequences. Functional pathways and genes were annotated against the Kyoto Encyclopedia of Genes and Genomes database [71].

### Statistical analysis

To test effects of compartment (air, leaf, root), time [seven sampling time points (TPs)], and treatment (control, N addition) on bacterial community diversity, composition, genomic traits, and predicted functional potentials, we used linear mixed-effects models with the lmer function (lme4) [72]. Time, compartment, and treatment were treated as fixed factors, and site was included as a random factor. To further assess effects of time and treatment within each compartment, separate mixed-effects models were fitted per compartment with time and treatment as fixed factors and site as a random factor.

To assess potential temporal autocorrelation, we fitted models with time as a fixed factor and site as a random factor and evaluated a first-order autoregressive correlation structure (AR(1), corAR1) [73]. Likelihood ratio tests indicated that including AR(1) neither improved model fit nor affected the significance of fixed factors (Supplementary Table S1). Residuals from models with time, compartment, and treatment as fixed factors and site as a random factor showed no apparent temporal structure (Supplementary Figs S4 and S5).

Relative abundances of bacterial phyla, families, and common OTUs were visualized with ggplot2 [74]. Permutational multivariate analysis of variance (PERMANOVA) was used to test effects of compartment, time, treatment, and their interactions on Bray–Curtis dissimilarities with the adonis function in vegan v2.6-4 [75]. Community compositional variation was visualized by principal coordinates (PCo) analysis using the pcoa function in ape v5.7-1 [76]. Mantel tests were used to evaluate correlations between temporal distance and community dissimilarity in vegan v2.6-4 [75, 77]. Bacterial indicator taxa associated with specific sampling times were identified using threshold indicator taxa analysis (TITAN) in the TITAN2 package v2.4.3 [29, 78]. For each taxon, the threshold (change point) was defined as the TP corresponding to the strongest change in relative abundance; only taxa with purity and reliability ≥0.95 were retained [78].

To examine temporal changes in community composition, genomic traits, and predicted functional potentials during the 7-month development of *P. massoniana* seedlings, we evaluated correlations with time using Spearman’s rank correlation and further tested these relationships with mixed-effects models including time as a fixed factor and site as a random factor (Supplementary Table S2). Model residuals were examined to verify assumptions (Supplementary Tables S2–S6; Supplementary Fig. S6). Differential functional analysis between environment-associated (air) and plant-associated (root, leaf) microbiomes was performed with DESeq2 v1.42.0 [79]. All statistical analyses were conducted in R v4.3.3 [80] unless stated otherwise.

## Results

**Figure 2:**
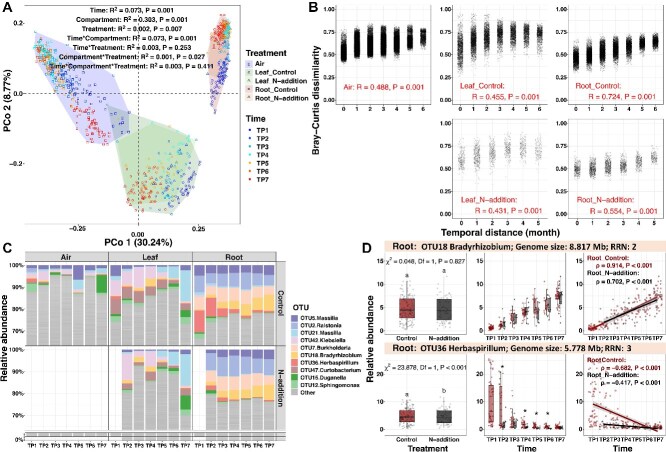
Temporal dynamics of bacterial communities in the roots and leaves of developing *Pinus* seedlings and the surrounding air; (A) principal coordinates (PCo) analysis of Bray–Curtis dissimilarities with permutational multivariate analysis of variance (PERMANOVA), showing significant effects of time, compartment, treatment, and their interactions on community composition; (B**)** Mantel tests assessing the association between temporal distance and Bray–Curtis dissimilarity indicate significant correlations in air, leaves, and roots, under control and N addition treatments (Mantel statistic, R); (C) temporal changes in the relative abundance of bacterial OTUs at each time point (TP) in different compartments (air, leaf, and root) and treatments (control and N addition); (D) relative abundance of root *Bradyrhizobium* OTU18 and *Herbaspirillum* OTU36 at different times and under different treatments; the genome size and RRN for these OTUs were predicted by aligning 16S rRNA gene sequences to reference genomes in GTDB v202 and rrnDB v5.8; differences were tested with the Kruskal–Wallis test with Bonferroni-adjusted *P* values; different letters (*P* < .001) and asterisks (*P* < .05) indicate significant differences between treatments; trend lines were fitted with linear regression; Spearman’s rank correlation results (ρ, *P*) are shown; the gray area around the fitted line indicates the 95% confidence interval; *n* = 825 samples.

We detected 5189 bacterial OTUs and 22 archaeal OTUs (0–4 OTUs per sample, 0–111 reads per sample) from 825 samples, dominated by *Proteobacteria*, *Actinobacteriota*, *Bacteroidota*, *Planctomycetota*, and *Acidobacteriota* (Supplementary Fig. S7). Thus, the following analysis focused only on the bacterial community. PCo analysis followed by PERMANOVA detected significant associations of bacterial community composition with time (*R*^2^ = 0.073, *P* = .001), compartment (*R*^2^ = 0.303, *P* = .001), and N treatment (*R*^2^ = 0.002, *P* = .007), as well as significant interactions between time and compartment (*R*^2^ = 0.073, *P* = .001) and between compartment and N treatment (*R*^2^ = 0.001, *P* = .027) (Fig. 2A). Significant temporal changes in bacterial community composition (overall *P* = .001) were detected by Mantel tests in air (*R* = 0.488), roots under control (*R* = 0.724) and N addition (*R* = 0.554), and leaves under control (*R* = 0.455) and N addition (*R* = 0.431), with temporal changes being most pronounced in root-associated communities (Fig. 2B).

Bacterial Shannon diversity and OTU richness were significantly higher in air than in roots and leaves, and significantly higher in root than in leaf under control but not under N addition (Supplementary Fig. S8B and D). Significant temporal changes in bacterial Shannon diversity and OTU richness were detected in air negatively, but root and leaves positively under control but not under N addition (Supplementary Fig. S8A and C).

A total of 1514 compartment indicator taxa are detected, with 266 biased toward air, 910 toward leaf, and 338 toward root (Supplementary Fig. S9). The result of TITAN revealed 325 temporal indicator taxa in air, 47 in leaves, and 130 in roots were detected using TITAN (Supplementary Figs S10–S12). In roots, OTU18 (*Bradyrhizobium* sp.) increased significantly as seedlings developed, whereas OTU36 (*Herbaspirillum* sp.) decreased significantly (Fig. 2C and D; Supplementary Fig. S13). N addition had no significant effect on *Bradyrhizobium* OTU18 but significantly reduced the relative abundance of *Herbaspirillum* OTU36, weakening its overall decreasing trend over time; nevertheless, the decline remained statistically significant (Fig. 2C and D; Supplementary Fig. S13). Similar genus-level trends were observed, with *Bradyrhizobium* increasing and *Herbaspirillum* decreasing under both control and N addition as seedlings developed (Supplementary Fig. S14).

**Figure 3:**
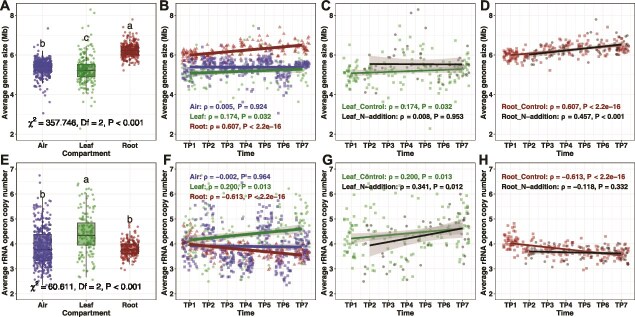
Temporal dynamics of bacterial community genomic traits in roots and leaves of developing *Pinus* seedlings and surrounding air; (A–H) bacterial genome size and rRNA operon copy number (RRN) were inferred by matching representative 16S rRNA gene sequences from each operational taxonomic unit (OTU) to the GTDB [64] and rrnDB [65]; bacterial average genome size and RRN for each sample were calculated as abundance-weighted means based on OTU read counts; besides OTUs, we also conducted genome size and RRN analyses based on bacterial amplicon sequence variant (ASV) data, as shown in Supplementary Fig. S15; (A) bacterial average genome size was significantly higher in roots than in both leaves and air, and higher in air than in leaves; different letters above the boxes indicate significant differences at *P* < .001 according to the Kruskal–Wallis test with Bonferroni correction; (B) bacterial average genome size increased over time in roots and leaves, and showed no significant temporal change in air; (C, D) temporal dynamics of bacterial average genome size in leaves and roots under different treatments (control and N addition); N addition did not affect the temporal dynamics of bacterial genome size; (E) bacterial average RRN was significantly higher in leaves than in both roots and air; (F) bacterial average RRN was significantly negatively correlated with time in roots, positively correlated with time in leaves, and showed no significant temporal change in air; (G, H) temporal dynamics of bacterial average RRN in leaves and roots under different treatments (control and N addition); N addition significantly affected the temporal dynamics of root bacterial average RRN, but not that in leaves; trend lines were fitted with linear regression; Spearman’s rank correlation results (ρ, *P*) are shown; the gray area around the fitted line indicates the 95% confidence interval; *n* = 825 samples.

To assess whether temporal changes in community composition were accompanied by shifts in bacterial genomic traits, we examined temporal dynamics in bacterial community genome size and RRN. Linear mixed-effects models revealed that the average bacterial community genome size was significantly affected by time and compartment, as well as by the interactions between time and compartment and between compartment and treatment (Supplementary Table S3). The average genome size was highest in roots (6.216 ± 0.024 Mb), followed by air (5.382 ± 0.021 Mb), and lowest in leaves (5.171 ± 0.054 Mb) (Fig. 3A). Average genome size was positively correlated with time in roots and leaves, with no significant temporal change in air (root: ρ = 0.607, *P* < .001; leaf: ρ = 0.174, *P* = .032; air: ρ = 0.005, *P* = .924) (Fig. 3B). N addition had little effect on genome size and did not alter its temporal dynamics in root, but led to nonsignificant temporal trends of average genome size in leaf (Fig. 3C and D; Supplementary Tables S3 and S5). Linear mixed-effects models also showed that the RRN of the bacterial community was significantly affected by treatment and by the interaction between time and compartment (Supplementary Table S3). The average RRN was higher in leaves (4.397 ± 0.066) than in roots (3.810 ± 0.023) and air (3.897 ± 0.040), and was not significantly different between root and air (Fig. 3E). Temporal trends in RRN differed among compartments, decreasing in roots, increasing in leaves, and remaining stable in air (root: ρ = −0.613, *P* < .001; leaf: ρ = 0.200, *P* = .013; air: ρ = −0.002, *P* = .964) (Fig. 3F). The time-by-treatment interaction on root RRN was significant: N addition decreased root RRN, leading to a nonsignificant decreasing trend over time (Fig. 3H; Supplementary Table S5). In contrast, N addition did not alter RRN temporal dynamics in leaf (Fig. 3G). ASV-based genomic trait estimates were largely consistent with OTU-based estimates, except for leaf-associated traits under the control treatment (which showed weak but significant changes when detected by OTUs but no significant changes when detected by ASVs) (Fig. 3; Supplementary Fig. S15).

Based on PICRUSt2 prediction, distinct temporal dynamics of predicted functional potential were observed in root, leaf, and air communities (Supplementary Figs S16–S18). In roots, the relative abundance of predicted pathways related to N fixation, amino acid, energy, carbohydrate, and nucleotide metabolism, terpenoids and polyketides, xenobiotic biodegradation and metabolism, and transcription increased over time, whereas pathways related to signal transduction, glycan biosynthesis and metabolism, transport and catabolism, and cell motility decreased (Supplementary Figs S16 and S18). In leaves, glycan biosynthesis and metabolism increased, while membrane transport decreased over time. In air, glycan biosynthesis and metabolism, signal transduction, and cell motility increased, while multiple metabolic functions decreased (Supplementary Figs S16 and S17A). Differential analyses further revealed compartment-specific enrichment of predicted functions: roots were enriched in xenobiotic biodegradation and metabolism, transport and catabolism, and diverse metabolic processes; leaves were mainly enriched in transcription and virus-related functions; air was enriched in signaling molecules and interaction, glycan biosynthesis and metabolism, transcription, translation, and chromosome-related functions (Supplementary Fig. S17B–D).

**Figure 4:**
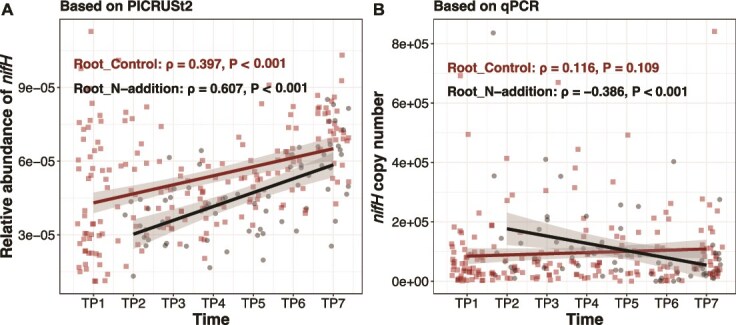
Temporal dynamics of predicted and measured *nifH* gene abundance in root-associated bacterial communities under control and N addition treatments; (A) predicted relative abundance of the *nifH* gene based on PICRUSt2 functional inference from 16S rRNA gene sequences; (B) the *nifH* gene copy number determined by quantitative real-time PCR (qPCR) using primers PolyF and PolyR; the *nifH* gene abundance was expressed as copy numbers per ng of DNA; trend lines were fitted with linear regression; Spearman’s rank correlation results (ρ, *P*) are shown; the gray area around the fitted line indicates the 95% confidence interval; *n* = 262 samples.

To assess the reliability of the predicted functional potentials, we quantified the *nifH* gene in root-associated bacteria using qPCR. PICRUSt2 predicted that the relative abundance of the *nifH* gene in root-associated bacteria was positively correlated with time under both control (ρ = 0.397, *P* < .001) and N addition treatments (ρ = 0.607, *P* < .001). However, qPCR results only partially supported this prediction: while a weak, non-significant increase was observed under the control treatment (ρ = 0.116, *P* = .109), *nifH* copy number declined significantly over time under N addition (ρ = −0.386, *P* < .001) (Fig. 4).

## Discussion

Our results revealed only partial support for the hypothesis regarding temporal dynamics of leaf bacterial average genome size (hypothesized to increase) and RRN (hypothesized to decrease). Over the 7-month seedling development period, leaf bacterial average genome size and RRN slightly increased when detected by OTUs but did not significantly change when detected by ASVs (Fig. 3; Supplementary Fig. S15C and G). In partial consistent with our observations, research on *Populus* reported a slight increase in the average genome size and decrease of RRN of leaf bacterial community over a 150-day period [32]. In contrast, a study on *Arabidopsis* over a 54-day period found decreases in both average genome size and RRN in leaf bacterial communities [32]. Differences between *Populus*, *Arabidopsis*, and our results may be attributed to factors such as experimental duration, leaf characteristics, host traits (herbaceous vs. woody), experimental conditions (indoor vs. field), and bacterial community profiling approach. Together, these comparisons highlight that the temporal dynamics of leaf-associated bacterial genomic traits are influenced by host traits and study context, and they underscore the need for similar host plant systems and long-term studies to establish general patterns. Our findings from the needle leaves of *P. massoniana* provide an important reference from a woody species, broadening the current understanding of leaf microbiome trait dynamics.

In contrast to leaves, root bacterial genomic traits displayed distinct and strong temporal dynamics, with increasing average genome size and decreasing RRN over time, regardless of OTUs or ASVs-based approach (Fig. 3; Supplementary Fig. S15D and H), fully consistent with our hypothesis. While Ortiz-Alvarez *et al*. [32] synthesized evidence for changes in bacterial genomic traits in leaves, flowers, and kelp lamina, the temporal dynamics of root-associated bacterial genomic traits have not been previously reported. Our findings provide novel evidence to bacterial genomic trait ecology by documenting root-associated temporal changes in *P. massoniana* seedlings. Previous studies on root microbiome succession in plants such as *Cupressus*, sorghum, soybean, and *Arabidopsis* have shown that microbial community composition shifts dramatically with plant development [31, 81–84], largely reflecting stage-specific changes in nutrient requirements and root exudation [85, 86]. Such successional dynamics in microbial community compositions are in line with our findings and provide additional ecological context, suggesting that the genomic trait change observed here is a community-aggregated trait of a restructuring root-associated microbiomes during seedling development. As the seedlings grow, the bacterial community in roots may become adapted to a more competitive environment, potentially shifting toward slower growth rates or more efficient resource utilization [20, 22, 87].

**Figure 5:**
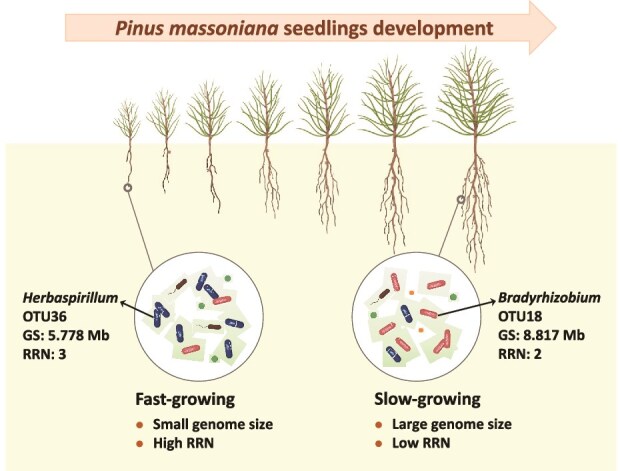
Conceptual model of the adaptive strategy of the root microbiome during the development of *Pinus massoniana* seedlings; GS, genome size; RRN, ribosomal RNA operon copy number.

The strong increase in average genome size and the decrease in RRN in root bacterial communities over time were unlikely to be caused by temporal dynamics in airborne bacterial communities, which showed no significant change over time (Fig. 3). Instead, the shifts in root bacterial genomic traits could be explained by the turnover of dominant bacterial taxa during the growth of *P. massoniana* seedlings (Fig. 2C). This includes an increase in the relative abundance of OTU18, a *Bradyrhizobium* with a relatively larger genome (8.817 Mb) and lower RRN (2), and a decrease in OTU36, a *Herbaspirillum* with a relatively smaller genome (5.778 Mb) and higher RRN (3) (Figs 2D and 5). Furthermore, based on the results of TITAN, we observed that the average genome size of temporally increased indicator OTUs was larger, whereas the average RRN of them was smaller, compared with decreased indicator OTUs (Supplementary Fig. S12). Together, these findings suggest that root bacterial communities in *P. massoniana* seedlings undergo shifts in genomic traits during the establishment phase, potentially favoring traits associated with competitive ability and resource efficiency rather than rapid growth.

Our hypothesis that N deposition would influence the temporal dynamics of plant-associated bacterial genomic traits was partially supported. N addition had little effect on the temporal dynamics of bacterial average genome size but significantly affected the temporal dynamics of bacterial RRN, especially in roots (Supplementary Table S5) [88]. These effects were consistent with taxonomic shifts in root-associated bacteria. N addition had little effect on root *Bradyrhizobium* OTU18 (large genome and low RRN) and did not affect its temporal dynamics (Fig. 2D), thereby contributing to the minimal overall effect of N on bacterial genome size. In contrast, N addition markedly reduced the relative abundance of root *Herbaspirillum* OTU36 (small genome and high RRN) immediately after the first N addition, a shift that paralleled the observed decline in root bacterial RRN (Figs 2D and 3H). The pronounced decline in RRN after the first N addition attenuated the temporal dynamics, rendering the overall trend nonsignificant under N treatment. In contrast, a prior study found that nitrogen deposition increased soil bacterial RRN in a Mediterranean grassland [89]. Given the differences in sample type (plant vs. soil) and ecosystem type (subtropical forest vs. Mediterranean grassland), we are far from a comprehensive understanding of these contrasting results.

In addition to shifts in taxonomic composition and genomic traits, we detected temporal changes in the predicted functional gene repertoire of root-associated bacterial communities. PICRUSt2 predicted increasing potentials for N fixation, transcriptional regulation, and metabolism of xenobiotics, terpenoids and polyketides, carbohydrates, and amino acids, while functions related to signal transduction declined over time (Supplementary Fig. S16). To provide an independent reference for these predictions, we quantified the *nifH* gene in root-associated bacteria by qPCR. Contrary to the PICRUSt2 trend of increasing *nifH* relative abundance across treatments, qPCR showed decreased *nifH* under N addition and no significant change under ambient conditions (Fig. 4). These discrepancies preclude firm inference about functional trajectories and underscore the need for caution when interpreting prediction-based functional profiles. Previous studies have demonstrated that qPCR measurements and PICRUSt-based functional estimates can exhibit correlations ranging from strongly positive to strongly negative [90]. Our findings reinforce growing concerns regarding the reliability of microbial gene function predictions based solely on computational estimates [91].

Our experimental design, in which seedlings were cultivated in sterilized soil:sand mixtures suspended above forest soil, offered advantages for investigating microbial dynamics during seedling establishment. By restricting colonization to aerial deposition [92], we tracked community assembly de novo without confounding effects from resident soil communities, generating clean temporal signals and enabling robust detection of genomic trait shifts within a single growing season. However, this approach has limitations. Aerial-mediated colonization differs from natural forest conditions, where soil-based inoculation predominates. In addition, the use of sterile substrate and the relatively short monitoring period limit extrapolation of our results to longer-term microbial dynamics in native soils. Despite rigorous sterilization protocols, the absence of longitudinal substrate monitoring prevents confirmation of sustained sterility. While continuous *in situ* monitoring would provide valuable context, such approaches face inherent challenges from environmental heterogeneity and rapid seedling turnover in natural settings [2].

We monitored only the first growing season of *P. massoniana* seedlings, a critical period during which plant–microbe interactions are established and genomic trait distributions can shift rapidly [2, 5]. Within a longer-term forest successional framework, the trait dynamics observed during this early stage likely represent initial conditions that shape microbiome evolution over extended timescales. Future work should extend to multiple *Pinus* and other tree species, longer monitoring periods, and different forest successional stages to test the generality of these dynamics. Nonetheless, our results demonstrate that detectable genomic trait shifts occur even during initial establishment, providing both a foundational framework and a methodological basis for such investigations. Despite being site-specific, our findings offer valuable insights into seedling survival in *Pinus*-dominated subtropical forests. Given the global distribution of *Pinus* and its roles in nutrient cycling, carbon storage, and forest structure [2], the observed microbial dynamics and responses to N addition may have broader implications for forest management across *Pinus* ecosystems.

Our genomic trait analysis focused on bacteria rather than fungi because fungal genomic trait reference resources remain limited. Currently, fewer than 100 entries for fungal rRNA operon copy number and ~2000 entries for fungal genome size are available, compared with 12 138 and 136 646 entries for bacteria, respectively [64, 65, 93, 94]. Recently, a pioneering study by Zhang *et al*. [95] showed that, on average, only ~3% of environmental fungal sequence reads per sample could be assigned trait data. Although this represents an important first step, the limited coverage of current fungal trait reference databases means that a substantial proportion of fungal taxa remains uncharacterized, introducing considerable uncertainty into trait-based inferences. As fungal genome resources continue to expand, future studies could also include fungal genomic traits to gain a more comprehensive understanding of temporal dynamics in seedling microbiomes. In addition, our bacterial genomic trait estimates were inferred from amplicon data rather than directly validated by metagenomics or qPCR. This predictive approach may introduce bias due to a lack of reference genomes for some OTUs. With most bacterial OTUs in our study (54.87%–94.14%, Supplementary Fig. S1) having matching reference genomes, future integration of metagenomic or genome-resolved metatranscriptomic analyses will provide a more direct assessment of microbial genomic trait dynamics.

## Conclusion

This study revealed the temporal dynamics of genomic traits of root- and leaf-associated microbiomes during the critical developmental phase of *P. massoniana* seedlings. While the genomic traits of leaf bacteria remained temporally static, those of root bacteria displayed pronounced temporal turnover, marked by progressive increases in average genome size and reductions in RRN. These shifts were decoupled from airborne microbiomes, emphasizing the primacy of host-regulated mechanisms over external environmental drivers in shaping microbial functional traits. The changes in genome size and RRN of root bacteria could result from taxonomic shifts during seedling development, i.e. an enrichment of taxa with larger genomes and lower RRN and a decline in those with smaller genomes and higher RRN. Nitrogen addition could affect the temporal dynamics of bacterial genomic traits in roots by accelerating the decline of taxa with higher RRN. Although these findings are derived from *P. massoniana*, similar temporal dynamics may also occur in other evergreen pines with comparable traits under similar environmental conditions.

## Supplementary Material

SupplementaryInformation_ycag018

## Data Availability

The raw sequence data reported in this article have been deposited in the Genome Sequence Archive [96] in National Genomics Data Center [97], China National Center for Bioinformation / Beijing Institute of Genomics, Chinese Academy of Sciences (GSA: CRA024361) that are publicly accessible at https://ngdc.cncb.ac.cn/gsa.

## Code availability

The source code implementing the analyses in this manuscript is available on Github (https://github.com/71suizhuguaibanzhuan/PinusBacterialData).
