# Supplementary material for: Root microbiome dynamics favor slow-growth strategies during Pinus seedling development
Source: ISME Commun. 2026 Jan 29;6(1):ycag018. doi: 10.1093/ismeco/ycag018 (PMC12915586; doi:10.1093/ismeco/ycag018)
Supplement: SupplementaryInformation_ycag018 [file supplementaryinformation_ycag018.docx]

**Root Microbiome Dynamics Favor Slow-Growth Strategy during *Pinus* Seedling Development**

**Running title:** Developing Seedling Favors Slow-Growth Microbiome

**Authors:** Chenyu Sun^1,2^, Cong Wang^2,3*^, Huanhuan Zhu^2,3^, Xiaoye Chen^2,3^, Peilin Chen^2,3^, Qiushi Li^2,3^, Zheng Gao^1^, Bo Yang^4^, Lei Chen^5^, Ningning Wang^5^, Liangdong Guo^2,3^, Cheng Gao^2,3*^

Supplementary information: Figure: 18, Table: 6.

*Corresponding author:

Cheng Gao, Tel: +86(10)64807024, E-mail: gaoc@im.ac.cn.

Cong Wang, Tel: +86(10)64807020, E-mail: wangc@im.ac.cn.

**Supplementary Fig. 1** Relative abundance of operational taxonomical units (OTUs) matched to bacterial genome size (**a**) and rRNA operon copy number (RRN) (**b**), based on alignments meeting sequence identity ≥ 95% and query coverage ≥ 95%. Cyan bars indicate OTUs retained for the genome size and RRN analyses (73.32-94.14% and 54.87-85.02%, respectively). Orange bars indicate excluded OTUs (identity < 95% and/or coverage < 95%). C, control (no N addition); N, N addition.

**Supplementary Fig. 2 Conservatism of genome size. a** Coefficient of variation (CV, %, left y-axis, blue bars) of reference genome sizes from the Genome Taxonomy Database (GTDB) at the genus level for taxa detected in this study. Red bars (right y-axis) indicate the relative abundances of the same taxa, ordered along the x-axis by decreasing CV. **b** Relative abundances of genera exhibiting low variability (CV < 0.25; blue) across compartments. Together, these results indicate that most dominant genera exhibit low variation in genome size estimates.

**Supplementary Fig. 3 Conservatism of rRNA operon copy number (RRN). a** Coefficient of variation (CV, %, left y-axis, blue bars) of reference RRN from the rrnDB at the genus level for taxa detected in this study. Red bars (right y-axis) indicate the relative abundances of the same taxa, ordered along the x-axis by decreasing CV. **b** Relative abundances of genera exhibiting low variability (CV < 0.25; blue) across compartments. Together, these results indicate that most dominant genera exhibit low variation in RRN estimates.

**Supplementary Fig. 4** Residuals of linear mixed-effects models (with site as a random factor and time, compartment, and treatment as fixed factors) for genomic traits and top 10 OTUs plotted against sampling time to evaluate potential temporal autocorrelation. No significant temporal structure was observed.

**Supplementary Fig. 5** Residuals of linear mixed-effects models (with site as a random factor and time, compartment, and treatment as fixed factors) for predicted gene functional potentials plotted against sampling time to evaluate potential temporal autocorrelation. No significant temporal structure was observed.

**Supplementary Fig. 6 Residual diagnostics for mixed-effects models (with site as a random factor and time as a fixed factors).** Shown are histograms (left) and normal Q-Q plots (right) of residuals from models predicting genome size and RRN across compartments (air, leaf, and root) and treatments (C: control; N: N-addition). These plots assess if residuals follow a normal distribution. The corresponding model results are provided in Supplementary Table 2 and 5.

**Supplementary** **Fig. 7** **Temporal dynamics of bacterial community in roots and leaves of *Pinus* seedlings and surrounding air.** **a**, **b** Temporal changes in relative abundance of bacterial phyla and genera at each time point (TP) in different compartments (air, leaf, and root) and treatments (Control and N addition). n = 825 samples.

**Supplementary** **Fig.** **8 Temporal dynamics of alpha diversity of bacterial communities in roots and leaves of *Pinus* seedlings and surrounding air.** **a-d** Shannon diversity and OTU richness were analyzed using linear mixed-effects models, with time, compartment, and treatment as fixed factors and site as a random factor. The detailed results of these analyses are provided in Supplementary Table 3. Shannon diversity and OTU richness of the bacterial community were highest in air, followed by roots, and lowest in leaves under the control treatment. C: control (without N addition), N: N addition. Differences were detected by the Kruskal-Wallis test with Bonferroni-adjusted P values. Trend lines were fitted with linear regression. Spearman’s rank correlation results (ρ, P) are shown. The grey area around the smooth line indicates the 95% confidence interval. n = 825 samples.

**Supplementary Fig. 9** **Ternary plots demonstrating the distribution of bacterial indicator taxa detected from the air, leaf, and root.** In total, 1,514 compartment-specific indicator taxa were identified, of which 266 were biased toward air, 910 toward leaf, and 338 toward root. Note the plot presented only significantly biased taxa (dots).

**Supplementary** **Fig. 10 Temporal dynamics of bacterial community and inferred genomic traits in surrounding air.** Significant temporal changes in relative abundances of bacterial OTUs of air samples were identified using TITAN. For each indicator OTU, the threshold was defined as the time point corresponding to the strongest abundance change. This included the corresponding genome size, and rRNA operon copy number (RRN) for each indicator OTU. Point size represents the relative abundance of OTUs. The red line indicates the average values of genome size and RRN of indicator OTUs with decreasing abundance (z−), while the blue line represents those with increasing abundance (z+). For each OTU, the phylum name is noted by color and filled symbols show declining abundance (z−), and open symbols show increasing abundance (z+). Only OTUs with both purity and reliability values ≥ 0.95 were retained as significant indicators. n = 357 samples.

**Supplementary** **Fig. 11 Temporal dynamics of bacterial community and inferred genomic traits in leaves of *Pinus* seedlings.** Significant temporal changes in relative abundances of bacterial OTUs of leaf samples were identified using TITAN. For each indicator OTU, the threshold was defined as the time point corresponding to the strongest abundance change. This included the corresponding genome size, and rRNA operon copy number (RRN) for each indicator OTU. Point size represents the relative abundance of OTUs. The red line indicates the average values of genome size and RRN of indicator OTUs with decreasing abundance (z−), while the blue line represents those with increasing abundance (z+). For each OTU, the phylum name is noted by color and filled symbols show declining abundance (z−), and open symbols show increasing abundance (z+). Only OTUs with both purity and reliability values ≥ 0.95 were retained as significant indicators. n = 193 samples (Control only).

**Supplementary** **Fig. 12** **Temporal dynamics of bacterial community and inferred genomic traits in roots of *Pinus* seedlings.** Significant temporal changes in relative abundances of bacterial OTUs of root samples were identified using TITAN. For each indicator OTU, the threshold was defined as the time point corresponding to the strongest abundance change. This included the corresponding genome size, and rRNA operon copy number (RRN) for each indicator OTU. Point size represents the relative abundance of OTUs. The red line indicates the average values of genome size and RRN of indicator OTUs with decreasing abundance (z−), while the blue line represents those with increasing abundance (z+). For each OTU, the phylum name is noted by color and filled symbols show declining abundance (z−), and open symbols show increasing abundance (z+). Only OTUs with both purity and reliability values ≥ 0.95 were retained as significant indicators. n = 192 samples (Control only).

**Supplementary** **Fig. 13** **Temporal dynamics of bacterial OTUs in roots of *Pinus* seedlings.** **a-j** Relative abundance of the top 10 root OTUs at different times and treatments. Bacterial genome size and rRNA operon copy number (RRN) were predicted by matching representative 16S rRNA gene sequences from each OTU to the GTDB v202 and the rrnDB v5.8. Differences were detected by the Kruskal-Wallis test with Bonferroni-adjusted P value. Different letters (P < 0.001) and asterisks (P < 0.05) indicate significant differences between treatments. Linear regression model with two-sided test was used for the statistical analysis. Spearman’s rank correlation results (ρ, P) are shown. The grey area around the smooth line indicates the 95% confidence interval. n = 262 samples.

**Supplementary** **Fig. 14** **Temporal dynamics of *Bradyrhizobium* and *Herbaspirillum* in roots of *Pinus* seedlings.** **a-b** Relative abundance of *Bradyrhizobium* and *Herbaspirillum* at different times and treatments. Differences were detected by the Kruskal-Wallis test with Bonferroni-adjusted P value. Different letters (P < 0.001) and asterisks (P < 0.05) indicate significant differences between treatments. Trend lines were fitted with linear regression. Spearman’s rank correlation results (ρ, P) are shown. The grey area around the smooth line indicates the 95% confidence interval. n = 262 samples.

**Supplementary Fig. 15 Temporal dynamics of bacterial community genomic traits in roots and leaves of developing *Pinus* seedlings and surrounding air.** **a-h** Bacterial genome size and rRNA operon copy number (RRN) were predicted by matching representative 16S rRNA gene sequences from each amplicon sequence variant (ASV) to the GTDB and rrnDB. Bacterial average genome size and RRN of each sample were calculated as abundance-weighted means based on ASV read counts. This ASV-based analysis provides supplementary evidence to the OTU-based results shown in Fig. 3.

**Supplementary Fig. 16 Correlation of bacterial community functions with time in roots and leaves of *Pinus* seedlings and surrounding air.** The temporal dynamics of bacterial functions are dependent on the compartment. Predicted functional potentials were screened based on the correlation (ρ value) with time in roots and their mean proportion. Functions with relatively high proportion and an ρ value greater than 0.380 were retained to identify those significantly associated with temporal changes. Predicted bacterial functions were inferred using PICRUSt2 based on OTU abundance and representative sequences. Nitrogen-fixing genes were identified by mapping predicted functional potentials to KEGG Orthologies (KOs), including *fix* and *nif* gene families. The three compartments are colored blue for air, forest green for leaf, and dark red for root. Trend lines were fitted with linear regression. Spearman’s rank correlation results (ρ, P) are shown. The grey area around the smooth line indicates the 95% confidence interval. n = 742 samples.

**Supplementary** **Fig. 17 Correlation of bacterial community functions with time and functional enrichment differences among compartments in *Pinus* seedlings and surrounding air. a** The heatmap shows the correlations between KEGG pathways in air, and root, and leaf of *Pinus* seedlings with time. The positive correlation between KEGG pathways and time is marked in blue, while the negative correlation between KEGG pathways and time is marked in red. KEGG pathways with significant differences are indicated by asterisks, P values are adjusted based on the false discovery rate (FDR). *P < 0.05, **P < 0.01, ***P < 0.001. Spearman’s rank correlation results (ρ) are shown. **b** Enrichments of functions in KEGG pathways for root and leaf bacterial communities. The functions of the root bacterial community were enriched in xenobiotic biodegradation and metabolism, transport and catabolism. The functions of the leaf bacterial community were enriched in transcription, information processing in viruses, viral protein families, and signaling molecules and interaction. **c** Enrichments of functions in KEGG pathways for root and air bacterial communities. The functions of the root bacterial community were enriched in xenobiotic biodegradation and metabolism, amino acid metabolism, transport and catabolism, cellular community-prokaryotes, membrane transport. The functions of the air bacterial community were enriched in signaling molecules and interaction, viral protein families, transcription, translation, and folding, sorting and degradation. **d** Enrichments of functions in KEGG pathways for leaf and air bacterial communities. The functions of the leaf bacterial community were enriched in viral protein families, xenobiotics biodegradation and metabolism, membrane transport, information processing in viruses, cellular community-prokaryotes, protein families-signaling and cellular processes. The functions of the air bacterial community were enriched in chromosome, signaling molecules and interaction, glycan biosynthesis and metabolism. n = 742 samples.

**Supplementary Fig. 18 Correlation of nitrogen-related genes of bacterial community with time in roots and leaves of *Pinus* seedlings and surrounding air.** Predicted bacterial functional potentials were inferred using PICRUSt2 based on OTU abundance and representative sequences. Nitrogen-related genes were identified by mapping predicted functional profiles to KOs. The three compartments are colored blue for air, forest green for leaf, and dark red for root. Trend lines were fitted with linear regression. Spearman’s rank correlation results (ρ, P) are shown. The grey area around the smooth line indicates the 95% confidence interval. n = 742 samples.

| **Supplementary Table 1** Linear mixed-effects models testing the effects of time (seven sampling time points) on bacterial genomic traits. | | | | | | | |
| --- | --- | --- | --- | --- | --- | --- | --- |
|  | | **Model 1** | | | **Model 2** | | **Model 1 vs Model 2** |
|  |  | **F** | **P** | **Phi** | **F** | **P** | **P** |
| **Genome size** | **Air** | 0.259 | 0.618 | -0.433 | 0.027 | 0.871 | 0.025 |
|  | **Leaf (Control)** | 0.372 | 0.552 | 0.407 | 0.401 | 0.538 | 0.717 |
|  | **Leaf (N addition)** | 0.008 | 0.929 | -0.286 | 0.001 | 0.976 | 0.167 |
|  | **Root (Control)** | 9.851 | 0.008 | 0.632 | 24.697 | <0.001 | 0.131 |
|  | **Root (N addition)** | 11.758 | 0.006 | 0.383 | 18.434 | 0.002 | 0.508 |
| **RRN** | **Air** | 0.101 | 0.755 | -0.427 | 0.0003 | 0.985 | 0.016 |
|  | **Leaf (Control)** | 1.232 | 0.287 | 0.664 | 2.333 | 0.151 | 0.1358 |
|  | **Leaf (N addition)** | 8.584 | 0.015 | -0.321 | 4.932 | 0.051 | 0.243 |
|  | **Root (Control)** | 16.778 | 0.0013 | 0.469 | 23.639 | <0.001 | 0.029 |
|  | **Root (N addition)** | 6.060 | 0.034 | -0.661 | 1.837 | 0.205 | 0.008 |
| Model 1: Includes a first-order autoregressive correlation structure (corAR1) to model temporal dependence within site. Time was treated as a fixed factor, and site was included as a random factor. Phi (ϕ, Model 1 only) : AR(1) autocorrelation coefficient, representing the degree of correlation between residuals from successive time points. Model 2: Identical fixed factor and random factor as Model 1, but no temporal autocorrelation. Model1 vs Model2 P (Likelihood Ratio Test): P-value from the likelihood ratio test. A significant P-value (<0.05) indicates temporal autocorrelation meaningfully improves model fit. | | | | | | | |

| **Supplementary Table 2** Results of linear mixed-effects models testing the effects of time (seven sampling time points) on bacterial community composition, genomic traits, and predicted gene functional potentials within each compartment (leaf, and root). | | | | | | | | | | |
| --- | --- | --- | --- | --- | --- | --- | --- | --- | --- | --- |
|  | | | **Time (control)** | | **Shapiro.test** | | **Time (N addition)** | | **Shapiro.test** | |
|  |  |  | **F** | **p** | **W** | **p** | **F** | **p** | **W** | **p** |
| **Root** | Genomic trait | Genome size | 94.106 | **<0.001** | 0.991 | 0.272 | 23.926 | **<0.001** | 0.948 | 0.006 |
|  |  | RRN | 95.510 | **<0.001** | 0.989 | 0.169 | 0.866 | 0.355 | 0.960 | 0.024 |
|  | OTU | OTU5 | 0.381 | 0.538 | 0.909 | <0.001 | 16.389 | **<0.001** | 0.959 | 0.023 |
|  |  | OTU2 | 3.482 | 0.064 | 0.932 | <0.001 | 1.974 | 0.166 | 0.904 | <0.001 |
|  |  | OTU21 | 19.600 | **<0.001** | 0.794 | <0.001 | 1.022 | 0.316 | 0.853 | <0.001 |
|  |  | OTU42 | 20.553 | **<0.001** | 0.467 | <0.001 | 5.923 | 0.018 | 0.709 | <0.001 |
|  |  | OTU7 | 1.081 | 0.300 | 0.771 | <0.001 | 0.947 | 0.336 | 0.897 | <0.001 |
|  |  | OTU18 | 777.930 | **<0.001** | 0.968 | <0.001 | 60.612 | **<0.001** | 0.991 | 0.914 |
|  |  | OTU36 | 91.228 | **<0.001** | 0.918 | <0.001 | 17.266 | **<0.001** | 0.738 | <0.001 |
|  |  | OTU47 | 4.137 | 0.043 | 0.725 | <0.001 | 2.431 | 0.124 | 0.494 | <0.001 |
|  |  | OTU15 | 33.173 | **<0.001** | 0.503 | <0.001 | 6.215 | 0.015 | 0.451 | <0.001 |
|  |  | OTU12 | 58.944 | **<0.001** | 0.754 | <0.001 | 29.184 | **<0.001** | 0.873 | <0.001 |
|  | Function | Nitrogen fixation | 117.310 | **<0.001** | 0.974 | 0.001 | 29.466 | **<0.001** | 0.987 | 0.687 |
|  |  | Amino acid metabolism | 138.930 | **<0.001** | 0.982 | 0.016 | 35.597 | **<0.001** | 0.978 | 0.262 |
|  |  | Transcription | 152.470 | **<0.001** | 0.959 | <0.001 | 1.713 | 0.196 | 0.838 | <0.001 |
|  |  | Energy metabolism | 133.320 | **<0.001** | 0.989 | 0.138 | 0.087 | 0.770 | 0.956 | 0.016 |
|  |  | M.terpenoids.polyketides | 82.414 | **<0.001** | 0.975 | 0.002 | 14.216 | **<0.001** | 0.871 | <0.001 |
|  |  | Carbohydrate.metabolism | 82.456 | **<0.001** | 0.990 | 0.174 | 15.284 | **<0.001** | 0.930 | 0.001 |
|  |  | Nucleotide Metabolism | 44.296 | **<0.001** | 0.994 | 0.578 | 8.614 | 0.005 | 0.940 | 0.002 |
|  |  | Xenobiotics.BD.metabolism | 33.291 | **<0.001** | 0.987 | 0.074 | 5.856 | 0.018 | 0.945 | 0.004 |
|  |  | Signal transduction | 145.610 | **<0.001** | 0.952 | <0.001 | 27.011 | **<0.001** | 0.956 | 0.015 |
|  |  | Glycan.biosyn.metabolism | 69.568 | **<0.001** | 0.992 | 0.347 | 8.839 | 0.004 | 0.938 | 0.002 |
|  |  | Transport and catabolism | 53.059 | **<0.001** | 0.960 | <0.001 | 4.279 | 0.042 | 0.924 | <0.001 |
|  |  | Cell motility | 51.975 | **<0.001** | 0.989 | 0.135 | 16.681 | **<0.001** | 0.941 | 0.002 |
| **Leaf** | Genomic  trait | Genome size | 2.318 | 0.130 | 0.952 | <0.001 | 0.003 | 0.958 | 0.904 | <0.001 |
|  |  | RRN | 5.344 | 0.022 | 0.986 | 0.114 | 4.299 | 0.044 | 0.953 | 0.032 |
|  | OTU | OTU5 | 17.241 | **<0.001** | 0.812 | <0.001 | 2.125 | 0.151 | 0.846 | <0.001 |
|  |  | OTU2 | 0.292 | 0.590 | 0.271 | <0.001 | 0.195 | 0.661 | 0.624 | <0.001 |
|  |  | OTU21 | 47.944 | **<0.001** | 0.749 | <0.001 | 27.957 | **<0.001** | 0.755 | <0.001 |
|  |  | OTU42 | 18.447 | **<0.001** | 0.646 | <0.001 | 9.184 | 0.004 | 0.619 | <0.001 |
|  |  | OTU7 | 0.061 | 0.806 | 0.347 | <0.001 | 0.127 | 0.723 | 0.678 | <0.001 |
|  |  | OTU18 | 0.100 | 0.752 | 0.385 | <0.001 | 14.363 | **<0.001** | 0.740 | <0.001 |
|  |  | OTU36 | 36.561 | **<0.001** | 0.719 | <0.001 | 0.005 | 0.943 | 0.526 | <0.001 |
|  |  | OTU47 | 16.248 | **<0.001** | 0.826 | <0.001 | <0.001 | 0.990 | 0.473 | <0.001 |
|  |  | OTU15 | 16.034 | **<0.001** | 0.618 | <0.001 | 5.314 | 0.025 | 0.550 | <0.001 |
|  |  | OTU12 | 0.023 | 0.879 | 0.865 | <0.001 | 14.867 | **<0.001** | 0.921 | 0.002 |
|  | Function | Nitrogen fixation | 0.818 | 0.367 | 0.937 | <0.001 | 5.339 | 0.025 | 0.964 | 0.101 |
|  |  | Amino acid metabolism | 1.443 | 0.232 | 0.982 | 0.040 | 0.094 | 0.760 | 0.943 | 0.013 |
|  |  | Transcription | 16.514 | **<0.001** | 0.960 | <0.001 | 1.433 | 0.237 | 0.965 | 0.111 |
|  |  | Energy metabolism | 5.311 | 0.023 | 0.985 | 0.092 | 1.602 | 0.212 | 0.970 | 0.183 |
|  |  | M.terpenoids.polyketides | 0.134 | 0.715 | 0.980 | 0.028 | 2.099 | 0.154 | 0.924 | 0.002 |
|  |  | Carbohydrate.metabolism | 9.002 | 0.003 | 0.985 | 0.096 | 9.153 | 0.004 | 0.982 | 0.576 |
|  |  | Nucleotide Metabolism | 1.353 | 0.247 | 0.897 | <0.001 | 2.288 | 0.137 | 0.974 | 0.292 |
|  |  | Xenobiotics.BD.metabolism | 0.397 | 0.530 | 0.978 | 0.014 | 6.233 | 0.016 | 0.974 | 0.278 |
|  |  | Signal transduction | 1.705 | 0.194 | 0.949 | <0.001 | 8.178 | 0.006 | 0.975 | 0.331 |
|  |  | Glycan.biosyn.metabolism | 29.258 | **<0.001** | 0.940 | <0.001 | 3.647 | 0.062 | 0.935 | 0.006 |
|  |  | Transport and catabolism | 16.008 | **<0.001** | 0.987 | 0.166 | 4.319 | 0.043 | 0.895 | <0.001 |
|  |  | Cell motility | 2.916 | 0.090 | 0.977 | 0.012 | 14.424 | **<0.001** | 0.992 | 0.972 |
| Time was included as fixed factors, and site was included as a random factor. Analyses were conducted separately for the control and N-addition treatments. Shapiro-Wilk tests were conducted to assess the normality of model residuals, with W and p values reported. | | | | | | | | | | |

|  | **Supplementary Table 3** Results of linear mixed-effects models testing the effects of time (seven sampling time points), compartment (air, leaf and root), and treatment (control and N addition) on bacterial community composition, and genomic traits. | | | | | | | | | | | | | | | | | | |
| --- | --- | --- | --- | --- | --- | --- | --- | --- | --- | --- | --- | --- | --- | --- | --- | --- | --- | --- | --- |
|  | | | **Time** | | **Compartment** | | **Treatment** | | **Time: Compartment** | | **Time: Treatment** | | **Compartment: Treatment** | | **Time: Compartment: Treatment** | | **Shapiro.test** | |  |
|  |  |  | **F** | **p** | **F** | **p** | **F** | **p** | **F** | **p** | **F** | **p** | **F** | **p** | **F** | **p** | **W** | **p** |  |
| **Genomic**  **trait** | | **Genome size** | 10.894 | **0.001** | 10.140 | **<0.001** | 2.590 | 0.108 | 11.323 | **<0.001** | 0.158 | 0.692 | 5.372 | 0.021 | 1.203 | 0.273 | 0.903 | <0.001 |  |
|  |  | **RRN** | 2.212 | 0.137 | 1.638 | 0.195 | 5.473 | 0.020 | 8.537 | **<0.001** | 3.421 | 0.065 | 0.076 | 0.783 | 0.004 | 0.950 | 0.973 | <0.001 |  |
| **OTU** | | **OTU5** | 2.042 | 0.153 | 19.591 | **<0.001** | 16.817 | **<0.001** | 12.663 | **<0.001** | 11.760 | **<0.001** | 0.162 | 0.688 | 0.193 | 0.661 | 0.814 | <0.001 |  |
|  |  | **OTU2** | 4.661 | 0.031 | 69.565 | **<0.001** | 2.260 | 0.133 | 1.717 | 0.180 | 0.074 | 0.786 | 1.456 | 0.228 | 0.0002 | 0.990 | 0.612 | <0.001 |  |
|  |  | **OTU21** | 104.775 | **<0.001** | 15.949 | **<0.001** | 11.478 | **<0.001** | 66.036 | **<0.001** | 9.684 | 0.002 | 5.268 | 0.022 | 5.770 | 0.017 | 0.611 | <0.001 |  |
|  |  | **OTU42** | 38.169 | **<0.001** | 34.065 | **<0.001** | 1.177 | 0.278 | 16.770 | **<0.001** | 0.982 | 0.322 | 2.415 | 0.121 | 1.993 | 0.158 | 0.496 | <0.001 |  |
|  |  | **OTU7** | 1.999 | 0.158 | 49.245 | **<0.001** | 0.364 | 0.546 | 1.300 | 0.273 | 0.139 | 0.710 | 0.470 | 0.493 | 0.149 | 0.699 | 0.605 | <0.001 |  |
|  |  | **OTU18** | 87.128 | **<0.001** | 20.545 | **<0.001** | 14.155 | **<0.001** | 250.692 | **<0.001** | 15.527 | **<0.001** | 6.170 | 0.013 | 3.166 | 0.076 | 0.639 | <0.001 |  |
|  |  | **OTU36** | 24.706 | **<0.001** | 90.786 | **<0.001** | 36.169 | **<0.001** | 45.231 | **<0.001** | 24.507 | **<0.001** | 20.130 | **<0.001** | 12.231 | **<0.001** | 0.606 | <0.001 |  |
|  |  | **OTU47** | 3.257 | 0.071 | 55.044 | **<0.001** | 13.851 | **<0.001** | 8.8578 | **<0.001** | 7.822 | 0.005 | 14.213 | **<0.001** | 8.107 | 0.005 | 0.502 | <0.001 |  |
|  |  | **OTU15** | 65.363 | **<0.001** | 23.232 | **<0.001** | 4.786 | 0.029 | 55.732 | **<0.001** | 9.121 | 0.003 | 8.873 | 0.003 | 13.065 | **<0.001** | 0.700 | <0.001 |  |
|  |  | **OTU12** | 4.180 | 0.041 | 3.322 | 0.037 | 18.610 | **<0.001** | 13.183 | **<0.001** | 11.791 | **<0.001** | 0.151 | 0.697 | 0.224 | 0.636 | 0.728 | <0.001 |  |
| **Alpha diversity** | | **Shannon index** | 0.356 | 0.551 | 180.374 | <0.001 | 3.868 | **0.050** | 17.796 | **<0.001** | 3.759 | **0.053** | 0.527 | 0.468 | 1.419 | 0.234 | 0.903 | <0.001 |  |
|  |  | **OTU richness** | 0.850 | 0.357 | 673.520 | <0.001 | 10.672 | **0.001** | 65.204 | **<0.001** | 7.675 | **0.006** | 0.757 | 0.384 | 0.034 | 0.854 | 0.980 | <0.001 |  |
| Time, compartment, and treatment were treated as fixed factors, and site was included as a random factor. Shapiro-Wilk tests were conducted to assess the normality of model residuals, with W and p values reported. Note: rRNA operon copy number (RRN). | | | | | | | | | | | | | | | | | | |  |

| **Supplementary Table 4** Results of linear mixed-effects models testing the effects of time (seven sampling time points), compartment (air, leaf and root), and treatment (control and N addition) on bacterial gene functions. | | | | | | | | | | | | | | | | |
| --- | --- | --- | --- | --- | --- | --- | --- | --- | --- | --- | --- | --- | --- | --- | --- | --- |
|  | **Time** | | **Compartment** | | **Treatment** | | **Time: Compartment** | | **Time: Treatment** | | **Compartment: Treatment** | | **Time: Compartment: Treatment** | | **Shapiro.test** | |
|  | **F** | **p** | **F** | **p** | **F** | **p** | **F** | **p** | **F** | **p** | **F** | **p** | **F** | **p** | **W** | **p** |
| **Nitrogen fixation** | 1.381 | 0.240 | 55.122 | **<0.001** | 1.751 | 0.186 | 41.503 | **<0.001** | 2.940 | 0.087 | 8.721 | 0.003 | 3.981 | 0.046 | 0.943 | <0.001 |
| **Amino acid metabolism** | 4.890 | 0.027 | 0.847 | 0.429 | 1.856 | 0.174 | 20.854 | **<0.001** | 1.119 | 0.291 | 0.949 | 0.330 | 1.078 | 0.299 | 0.908 | <0.001 |
| **Transcription** | 5.044 | 0.025 | 74.335 | **<0.001** | 2.040 | 0.154 | 21.014 | **<0.001** | 2.840 | 0.092 | 0.057 | 0.812 | 0.033 | 0.856 | 0.948 | <0.001 |
| **Energy metabolism** | 0.081 | 0.777 | 68.617 | **<0.001** | 13.161 | **<0.001** | 20.403 | **<0.001** | 11.976 | **<0.001** | 1.950 | 0.163 | 1.695 | 0.193 | 0.971 | <0.001 |
| **M.terpenoids.polyketides** | 0.013 | 0.908 | 16.960 | **<0.001** | 8.687 | 0.003 | 43.022 | **<0.001** | 3.673 | 0.056 | 7.427 | 0.007 | 5.134 | 0.024 | 0.966 | <0.001 |
| **Carbohydrate.metabolism** | 5.136 | 0.024 | 40.878 | **<0.001** | 4.394 | 0.036 | 34.363 | **<0.001** | 1.807 | 0.179 | 4.941 | 0.027 | 3.118 | 0.078 | 0.993 | <0.001 |
| **Nucleotide Metabolism** | 11.269 | **<0.001** | 75.2006 | **<0.001** | 0.119 | 0.730 | 2.358 | 0.095 | 0.0033 | 0.954 | 0.002 | 0.968 | 0.197 | 0.657 | 0.954 | <0.001 |
| **Xenobiotics.BD.metabolism** | 0.209 | 0.648 | 34.129 | **<0.001** | 3.466 | 0.063 | 18.428 | **<0.001** | 2.043 | 0.153 | 1.026 | 0.311 | 1.191 | 0.276 | 0.980 | <0.001 |
| **Signal transduction** | 4.334 | 0.038 | 40.258 | **<0.001** | 7.525 | 0.006 | 43.987 | **<0.001** | 7.193 | 0.007 | 8.163 | 0.004 | 5.619 | 0.018 | 0.977 | <0.001 |
| **Glycan.biosyn.metabolism** | 6.256 | 0.013 | 26.590 | **<0.001** | 0.360 | 0.549 | 66.766 | **<0.001** | 0.013 | 0.910 | 0.206 | 0.650 | 0.207 | 0.649 | 0.990 | <0.001 |
| **Transport and catabolism** | 13.569 | **<0.001** | 79.102 | **<0.001** | 0.479 | 0.489 | 51.806 | **<0.001** | 1.020 | 0.313 | 0.004 | 0.952 | 0.031 | 0.861 | 0.974 | <0.001 |
| **Cell motility** | 12.141 | **<0.001** | 22.420 | **<0.001** | 11.579 | **<0.001** | 41.593 | **<0.001** | 10.274 | 0.001 | 10.652 | 0.001 | 13.102 | **<0.001** | 0.983 | <0.001 |
| Time, compartment, and treatment were treated as fixed factors, and site was included as a random factor. Shapiro-Wilk tests were conducted to assess the normality of model residuals, with W and p values reported. Note: Metabolism of terpenoids and polyketides (M.terpenoids.polyketides), Xenobiotics biodegradation and metabolism (Xenobiotics.BD.metabolism), Glycan biosynthesis and metabolism (Glycan.biosyn.metabolism). | | | | | | | | | | | | | | | | |

| **Supplementary Table 5** Results of linear mixed-effects models testing the effects of treatment (control and N addition) and time (seven sampling time points) on bacterial community composition, and genomic traits within each compartment (air, leaf, and root). | | | | | | | | | | |
| --- | --- | --- | --- | --- | --- | --- | --- | --- | --- | --- |
|  | | | **Time** | | **Treatment** | | **Time: Treatment** | | **Shapiro.test** | |
|  |  |  | **F** | **p** | **F** | **p** | **F** | **p** | **W** | **p** |
| **Root** | **Genomic**  **trait** | **Genome size** | 78.471 | **<0.001** | 1.567 | 0.212 | 1.602 | 0.207 | 0.978 | <0.001 |
|  |  | **RRN** | 25.815 | **<0.001** | 15.807 | **<0.001** | 11.599 | **<0.001** | 0.996 | 0.771 |
|  | **OTU** | **OTU5** | 3.921 | 0.049 | 10.042 | 0.002 | 6.737 | 0.0099 | 0.913 | <0.001 |
|  |  | **OTU2** | 4.007 | 0.046 | 2.502 | 0.115 | 0.151 | 0.698 | 0.929 | <0.001 |
|  |  | **OTU21** | 4.085 | 0.044 | 14.033 | **0.0002** | 5.868 | 0.016 | 0.776 | <0.001 |
|  |  | **OTU42** | 5.892 | 0.016 | 5.343 | 0.022 | 4.405 | 0.037 | 0.438 | <0.001 |
|  |  | **OTU7** | 1.523 | 0.218 | 0.214 | 0.644 | 0.060 | 0.806 | 0.793 | <0.001 |
|  |  | **OTU18** | 385.041 | **<0.001** | 0.694 | 0.406 | 1.965 | 0.162 | 0.979 | 0.001 |
|  |  | **OTU36** | 32.265 | **<0.001** | 23.898 | **<0.001** | 15.320 | **0.0001** | 0.891 | <0.001 |
|  |  | **OTU47** | 5.354 | 0.021 | 0.032 | 0.858 | 0.318 | 0.573 | 0.678 | <0.001 |
|  |  | **OTU15** | 28.801 | **<0.001** | 4.806 | 0.029 | 2.700 | 0.102 | 0.464 | <0.001 |
|  |  | **OTU12** | 23.460 | **<0.001** | 13.407 | **0.0003** | 8.116 | 0.005 | 0.723 | <0.001 |
| **Leaf** | **Genomic**  **trait** | **Genome size** | 0.267 | 0.606 | 3.193 | 0.075 | 0.490 | 0.485 | 0.939 | <0.001 |
|  |  | **RRN** | 9.328 | 0.003 | 2.047 | 0.154 | 1.111 | 0.293 | 0.987 | 0.050 |
|  | **OTU** | **OTU5** | 3.345 | 0.069 | 7.493 | 0.007 | 5.258 | 0.023 | 0.807 | <0.001 |
|  |  | **OTU2** | 0.000 | 0.997 | 0.422 | 0.517 | 0.277 | 0.599 | 0.326 | <0.001 |
|  |  | **OTU21** | 64.583 | **<0.001** | 4.403 | 0.037 | 4.178 | 0.042 | 0.754 | <0.001 |
|  |  | **OTU42** | 22.141 | **<0.001** | 1.101 | 0.295 | 0.920 | 0.339 | 0.647 | <0.001 |
|  |  | **OTU7** | 0.0014 | 0.971 | 0.003 | 0.957 | 0.080 | 0.778 | 0.374 | <0.001 |
|  |  | **OTU18** | 5.157 | 0.024 | 7.782 | 0.006 | 6.497 | 0.012 | 0.449 | <0.001 |
|  |  | **OTU36** | 6.169 | 0.014 | 12.136 | **0.0006** | 11.166 | **0.001** | 0.695 | <0.001 |
|  |  | **OTU47** | 2.831 | 0.094 | 6.281 | 0.013 | 3.645 | 0.058 | 0.767 | <0.001 |
|  |  | **OTU15** | 31.345 | **<0.001** | 4.926 | 0.028 | 8.677 | 0.004 | 0.559 | <0.001 |
|  |  | **OTU12** | 4.258 | 0.040 | 6.057 | 0.015 | 3.855 | 0.051 | 0.872 | <0.001 |
| **Air** | **Genomic**  **trait** | **Genome size** | 0.084 | 0.772 |  |  |  |  | 0.910 | <0.001 |
|  |  | **RRN** | 0.134 | 0.714 |  |  |  |  | 0.970 | <0.001 |
|  | **OTU** | **OTU5** | 5.978 | 0.015 |  |  |  |  | 0.719 | <0.001 |
|  |  | **OTU2** | 4.54 | 0.034 |  |  |  |  | 0.091 | <0.001 |
|  |  | **OTU21** | 27.592 | **<0.001** |  |  |  |  | 0.559 | <0.001 |
|  |  | **OTU42** | 14.65 | **0.0002** |  |  |  |  | 0.432 | <0.001 |
|  |  | **OTU7** | 15.695 | **<0.001** |  |  |  |  | 0.548 | <0.001 |
|  |  | **OTU18** | 57.633 | **<0.001** |  |  |  |  | 0.823 | <0.001 |
|  |  | **OTU36** | 6.303 | 0.013 |  |  |  |  | 0.140 | <0.001 |
|  |  | **OTU47** | 20.298 | **<0.001** |  |  |  |  | 0.498 | <0.001 |
|  |  | **OTU15** | 122.92 | **<0.001** |  |  |  |  | 0.848 | <0.001 |
|  |  | **OTU12** | 41.165 | **<0.001** |  |  |  |  | 0.556 | <0.001 |
| Time and treatment were included as fixed factors, and site was included as a random factor. Shapiro-Wilk tests were conducted to assess the normality of model residuals, with W and p values reported. Note: rRNA operon copy number (RRN). | | | | | | | | | | |

| **Supplementary Table 6** Results of linear mixed-effects models testing the effects of treatment (control and N addition) and time (seven sampling time points) on bacterial community gene functions within each compartment (air, leaf, and root). | | | | | | | | | |
| --- | --- | --- | --- | --- | --- | --- | --- | --- | --- |
|  | | **Time** | | **Treatment** | | **Time: Treatment** | | **Shapiro.test** | |
|  |  | **F** | **p** | **F** | **p** | **F** | **p** |  |  |
| **Root** | **Nitrogen fixation** | 90.170 | **<0.001** | 1.982 | 0.160 | 0.003 | 0.954 | 0.990 | 0.082 |
|  | **Amino acid metabolism** | 108.293 | **<0.001** | 0.029 | 0.866 | 0.130 | 0.718 | 0.985 | 0.008 |
|  | **Transcription** | 44.715 | **<0.001** | 13.633 | **0.0003** | 11.312 | **0.0009** | 0.920 | <0.001 |
|  | **Energy metabolism** | 34.828 | **<0.001** | 19.130 | **<0.001** | 17.791 | **<0.001** | 0.990 | 0.075 |
|  | **M.terpenoids.polyketides** | 66.855 | **<0.001** | 0.222 | 0.638 | 1.081 | 0.299 | 0.947 | <0.001 |
|  | **Carbohydrate.metabolism** | 70.051 | **<0.001** | 0.551 | 0.459 | 1.274 | 0.260 | 0.973 | <0.001 |
|  | **Nucleotide Metabolism** | 29.362 | **<0.001** | 0.600 | 0.807 | 0.340 | 0.560 | 0.990 | 0.078 |
|  | **Xenobiotics.BD.metabolism** | 21.684 | **<0.001** | 0.466 | 0.496 | 0.050 | 0.824 | 0.992 | 0.166 |
|  | **Signal transduction** | 105.082 | **<0.001** | 0.341 | 0.560 | 0.000 | 0.996 | 0.956 | <0.001 |
|  | **Glycan.biosyn.metabolism** | 37.555 | **<0.001** | 0.0006 | 0.980 | 0.162 | 0.687 | 0.982 | 0.002 |
|  | **Transport and catabolism** | 25.268 | **<0.001** | 0.479 | 0.490 | 1.668 | 0.198 | 0.964 | <0.001 |
|  | **Cell motility** | 46.853 | **<0.001** | 0.0015 | 0.969 | 0.340 | 0.560 | 0.992 | 0.144 |
| **Leaf** | **Nitrogen fixation** | 2.082 | 0.151 | 6.593 | 0.011 | 5.084 | 0.025 | 0.956 | <0.001 |
|  | **Amino acid metabolism** | 0.017 | 0.897 | 0.845 | 0.359 | 0.669 | 0.414 | 0.977 | 0.002 |
|  | **Transcription** | 8.125 | 0.005 | 0.080 | 0.777 | 0.425 | 0.515 | 0.970 | <0.001 |
|  | **Energy metabolism** | 0.029 | 0.865 | 3.937 | 0.049 | 3.406 | 0.066 | 0.988 | 0.094 |
|  | **M.terpenoids.polyketides** | 2.562 | 0.111 | 5.953 | 0.016 | 3.034 | 0.083 | 0.973 | 0.001 |
|  | **Carbohydrate.metabolism** | 16.391 | **<0.001** | 3.989 | 0.047 | 1.869 | 0.173 | 0.990 | 0.158 |
|  | **Nucleotide Metabolism** | 2.855 | 0.093 | 0.268 | 0.606 | 0.338 | 0.562 | 0.907 | <0.001 |
|  | **Xenobiotics.BD.metabolism** | 4.762 | 0.030 | 3.191 | 0.076 | 2.414 | 0.122 | 0.983 | 0.014 |
|  | **Signal transduction** | 11.466 | **0.0009** | 5.835 | 0.0167 | 4.540 | 0.034 | 0.965 | <0.001 |
|  | **Glycan.biosyn.metabolism** | 17.219 | **<0.001** | 0.873 | 0.351 | 0.310 | 0.578 | 0.947 | <0.001 |
|  | **Transport and catabolism** | 14.805 | **0.0002** | 0.259 | 0.611 | 0.296 | 0.587 | 0.972 | <0.001 |
|  | **Cell motility** | 20.438 | **<0.001** | 8.154 | 0.005 | 8.480 | 0.004 | 0.987 | 0.064 |
| **Air** | **Nitrogen fixation** | 15.872 | **<0.001** |  |  |  |  | 0.922 | <0.001 |
|  | **Amino acid metabolism** | 5.603 | 0.018 |  |  |  |  | 0.827 | <0.001 |
|  | **Transcription** | 5.939 | 0.015 |  |  |  |  | 0.935 | <0.001 |
|  | **Energy metabolism** | 9.035 | 0.003 |  |  |  |  | 0.975 | <0.001 |
|  | **M.terpenoids.polyketides** | 36.03 | **<0.001** |  |  |  |  | 0.980 | <0.001 |
|  | **Carbohydrate.metabolism** | 19.654 | **<0.001** |  |  |  |  | 0.993 | 0.106 |
|  | **Nucleotide Metabolism** | 0.972 | 0.325 |  |  |  |  | 0.981 | <0.001 |
|  | **Xenobiotics.BD.metabolism** | 8.646 | 0.003 |  |  |  |  | 0.963 | <0.001 |
|  | **Signal transduction** | 25.28 | **<0.001** |  |  |  |  | 0.989 | 0.008 |
|  | **Glycan.biosyn.metabolism** | 100.58 | **<0.001** |  |  |  |  | 0.993 | 0.082 |
|  | **Transport and catabolism** | 81.766 | **<0.001** |  |  |  |  | 0.979 | <0.001 |
|  | **Cell motility** | 22.219 | **<0.001** |  |  |  |  | 0.985 | 0.001 |
| Time and treatment were included as fixed factors, and site was included as a random effect. Shapiro-Wilk tests were conducted to assess the normality of model residuals, with W and p values reported.  Note: Metabolism of terpenoids and polyketides (M.terpenoids.polyketides), Xenobiotics biodegradation and metabolism (Xenobiotics.BD.metabolism), Glycan biosynthesis and metabolism (Glycan.biosyn.metabolism). | | | | | | | | | |
